# Natural course of the nodular bronchiectatic form of *Mycobacterium Avium* complex lung disease: Long-term radiologic change without treatment

**DOI:** 10.1371/journal.pone.0185774

**Published:** 2017-10-02

**Authors:** Tae Yun Park, Semin Chong, Jae-Woo Jung, In Won Park, Byoung Whui Choi, Changwon Lim, Chang Un Lee, Yang Soo Kim, Hye Won Choi, Jae Chol Choi

**Affiliations:** 1 Division of Pulmonary Medicine, Department of Internal Medicine, Chung-Ang University, Chung-Ang University College of Medicine, Seoul, Korea; 2 Department of Radiology, Chung-Ang University Hospital, Chung-Ang University College of Medicine, Seoul, Korea; 3 Department of Applied Statistics, Chung-Ang University, Seoul, Korea; National Institute of Infectious Diseases, JAPAN

## Abstract

**Background/Purpose:**

Although the incidence of *Mycobacterium avium* complex (MAC) lung disease is increasing, the long-term natural course of the nodular bronchiectatic form of MAC lung disease is not well described. The objective of our study is to evaluate long-term radiologic changes in untreated MAC lung disease by analyzing serial chest computed tomography (CT) scan findings.

**Methods:**

Of 104 patients with MAC lung disease, we selected 40 untreated nodular bronchiectatic MAC patients who underwent serial chest CTs without treatment for at least four years (mean = 6.23 years). Majority of patients have minimal symptoms. Two chest radiologists retrospectively reviewed initial and final chest CT scans. Each chest CT scan was scored for presence and extent of bronchiectasis, cellular bronchiolitis, consolidation, cavity, and nodule (maximum score: 30).

**Results:**

Of 40 patients, 39 (97.5%) experienced a significant increase in overall CT score (overall difference = 4.89, p<0.001). On repeated measure analysis of variance analysis, cavity yielded the largest increase compared with cellular bronchiolitis (p = 0.013), nodule (p<0.001), and consolidation (p = 0.004). However, there was no significant difference in mean score change between cavity and bronchiectasis (p = 0.073). In analysis between radiologic parameters and the absolute number of involved segments, bronchiectasis showed most significant change compared with nodule (p<0.001) and consolidation (p<0.001).

**Conclusions:**

Most untreated nodular bronchiectatic MAC lung disease cases showed radiologic deterioration over long-term observation periods when we compared serial chest CT scans. Careful monitoring of MAC lung disease with serial chest CT scan can be beneficial in these untreated patients.

## Introduction

Non-tuberculous mycobacteria (NTM) are opportunistic and environmental pathogens. There has been a recent growing interest in NTM lung disease because of rapidly increasing worldwide prevalence. According to a survey from the United States of America (USA), NTM infection prevalence increased from 1.4 to 6.6 per 100,000 persons.[1] In Korea, the number of newly diagnosed NTM lung disease patients has been growing in recent years.[2–6]

Among the numerous NTM species, *Mycobacterium avium* complex (MAC) is the most globally common pathogen that causes lung disease. MAC lung disease has two major clinical forms: a fibrocavitary form that often affects older male smokers and a nodular bronchiectatic (BE) form that typically occurs in middle-aged non-smoking women. While the fibrocavitary form progresses extensively within a few years, the nodular BE form tends to progress slowly over a long period.[7–9] Therefore, treatment consensus varies according to clinical form. Contrary to the fibrocavitary form, which requires immediate treatment after diagnosis, the nodular BE form can be managed more cautiously by observation. Furthermore, in consideration of potential drug side effects and low rates of initial treatment success, immediate treatment of MAC lung disease may be ineffective, especially for the nodular BE form. [3, 8, 10–12]

Although the nodular BE form of MAC lung disease progresses slowly, it causes substantial deterioration over time.[13, 14] However, appropriate treatment timing and criteria are not established for these long follow-up periods.[15] In this respect, it is very important to understand the “outcome” of long-term prognosis for the nodular BE form of MAC lung disease without treatment. Therefore, we performed this study to identify the long-term natural course of the nodular BE form of MAC lung disease without treatment and to determine significant radiological changes by analyzing serial chest computed tomography (CT) findings.

## Materials and methods

### Study design and patients

**Fig 1:**
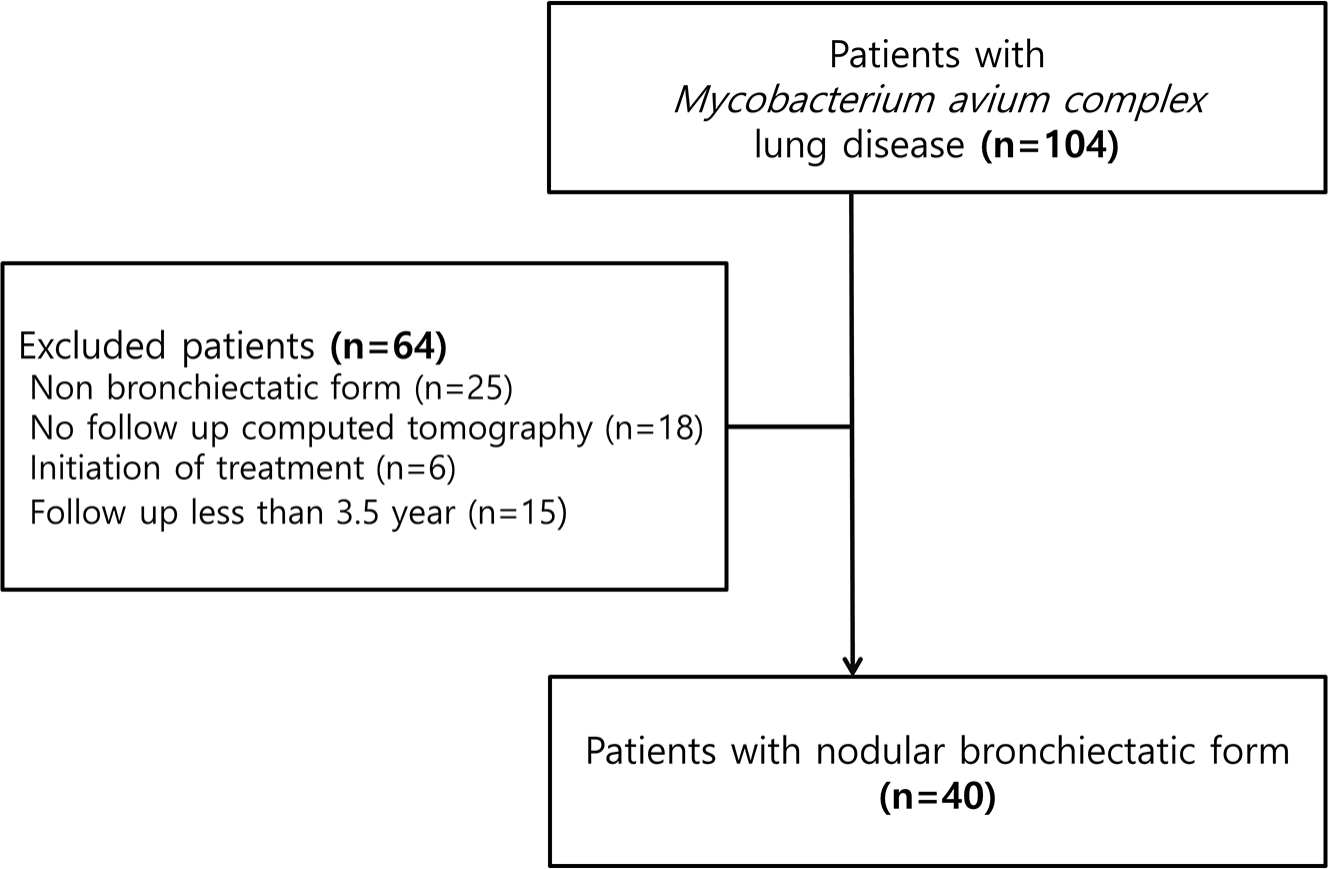
Flow chart of patients with MAC lung disease.

We conducted a retrospective cohort study from January 2005 to May 2012 in a single tertiary referral hospital. We included patients with the nodular BE form of MAC lung disease who did not receive treatment due to minimal symptom but who underwent serial CT scans at least four-year intervals. We defined these group as stationary group. Nodular BE form was defined when a patient had bronchiectasis and small nodules in the right middle lobe and lingular division of the left upper lobe, irrespective of cavity presence. During this period, 104 patients were diagnosed with MAC lung disease based on the 2007 American Thoracic society (ATS) guidelines.[8] Among these 104 patients, we excluded patients with non-nodular BE (n = 25), those who were lost to follow-up (n = 18), who started treatment within 4 years interval (n = 6), and who did not undergo serial chest CT scans at least four-year intervals (n = 15). Finally, 40 patients were included in this study (Fig 1). Chung-Ang University Hostpital Institutional Review Board approved this retrospective cohort study and waived written informed consent (IRB No: 1610-005-259).

### Data collection

Clinical factors including age, sex, body mass index (BMI), symptoms, and past medical history were collected from the electronic medical-record system. We evaluated radiological changes between the initial chest CT and the last follow-up chest CT before treatment. During the study period, we used different CT scanners due to long-term follow-up periods. All CT images were obtained from the lung apices to bases using both high-spatial-frequency and standard reconstruction algorithms with the following parameters: 120–140 kVp; 100–200 mA; reconstruction interval, 1–2.5 mm; section thickness, 1–8 mm for axial images and 1–2.5 mm for coronal images. All images were reviewed on both mediastinal (width: 350 HU, level: 50 HU) and lung (width: 1,500 HU, level: -700 HU) windows with a picture archiving and communication system (Marosis; Infinitt Co., Ltd., Seoul, Korea), which displayed all image data on two monitors (1,536 × 2,048 matrix, 8-bit viewable gray scale and 60-ft-lambert luminescence). Two thoracic radiologists with more than 10 years of experience who were blinded to clinical data reviewed all CT images in a random order.

**Fig 2:**
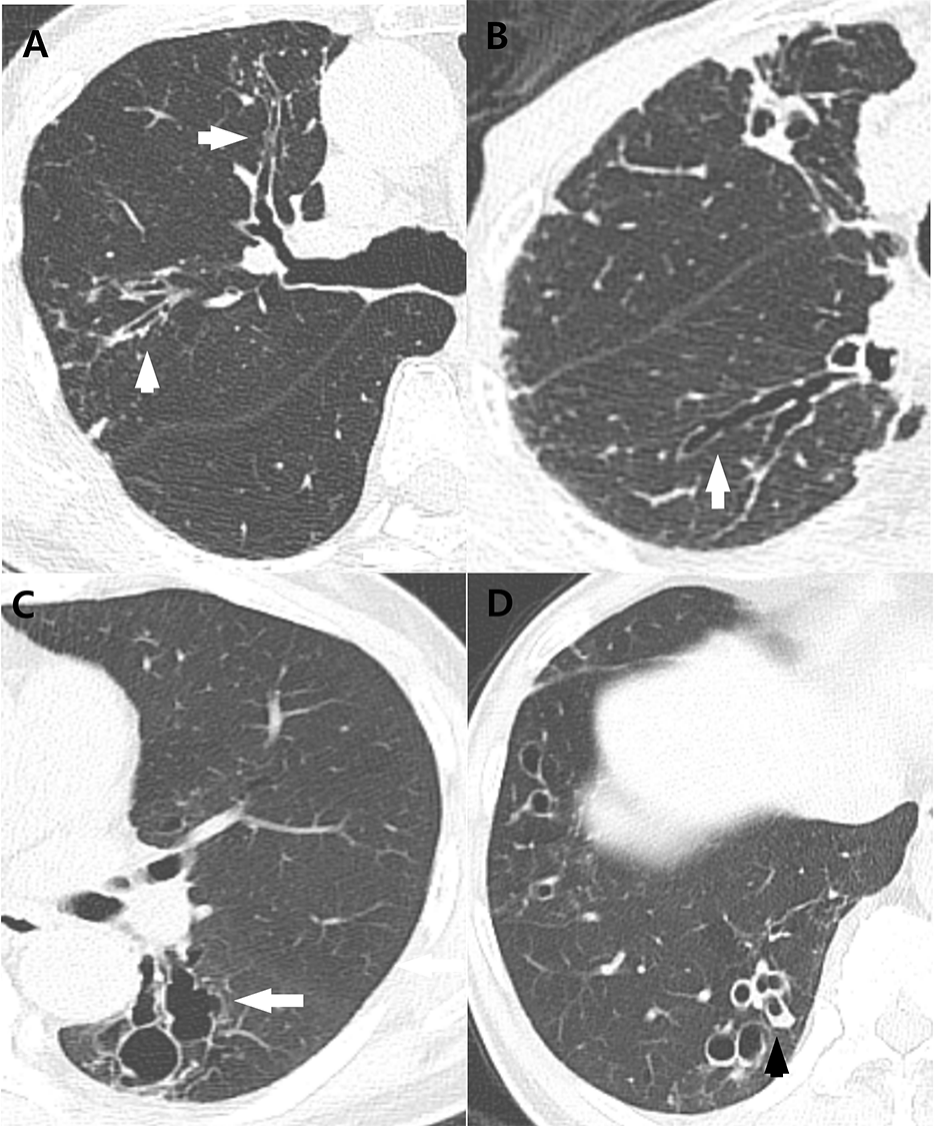
Examples of bronchiectasis to CT scoring of MAC lung disease. (A) Mild bronchiectasis in anterior and posterior segments of right upper lobe of 67-year-old man, scored as 1 point, with bronchus diameter greater than adjacent vessel diameter (arrows). (B) Moderate bronchiectasis in superior segment of right lower lobe of 86-year-old woman, scored as 2 points, with bronchus diameter two to three times vessel diameter (arrow). (C) Severe bronchiectasis in superior segment of left lower lobe of 66-year-old man, scored as 3 points, with bronchus diameter greater than three times vessel diameter (arrow). (D) Mucus plugging associated with severe bronchiectasis in posterior basal segment of right lower lobe of 66-year-old man (arrow).

**Fig 3:**
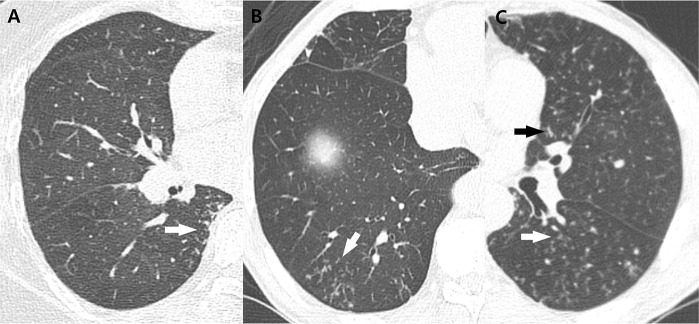
Examples of cellular bronchiolitis to CT scoring of MAC lung disease. (A) Mild cellular bronchiolitis in superior segment of right lower lobe of 67-year-old woman, scored as 1 point, manifesting as tree-in-bud patterns in the peripheral lung, 1 cm from the pleura (arrow). (B) Moderate cellular bronchiolitis in posterior basal segment of right lower lobe of 73-year-old man, scored as 2 points, showing centrilobular nodules or tree-in-bud patterns involved greater than 1–3 cm from the pleura (arrow). (C) Severe cellular bronchiolitis in superior segment of left lower lobe and lingular segment of left upper lobe of 59-year-old man, scored as 3 points, showing centrilobular nodules or tree-in-bud patterns extending to the central lung (arrows).

Each lung lobe was evaluated for five major parenchymal abnormalities, namely, bronchiectasis, cellular (or inflammatory) bronchiolitis, nodules 10–30 mm in diameter, consolidation, and cavities, which is consistent with previous study protocols.[15, 16] Bronchiectasis was defined as the bronchial dilatation larger than the diameter of the accompanying pulmonary artery. Mucus plugging was associated with bronchial dilatation and was defined as a lesion with linear or branching pattern within the proximal bronchus (lobar, segmental, or subsegmental bronchus). Cellular bronchiolitis was defined as either centrilobular nodules less than 10 mm in size or branching nodular structures or tree-in-bud pattern on CT scans. Airspace consolidation included all types of distributions (i.e. lobular, subsegmental or segmental etc.). Bronchiectasis, cellular bronchiolitis, and cavities were evaluated according to the following subcategories: bronchiectasis was subcategorized into severity, extent, and mucus-plugging; cellular bronchiolitis was subcategorized into severity and extent; and cavity was subcategorized into diameter, wall thickness, and extent. The examples of bronchiectasis and cellular bronchiolitis were described in Figs 2 and 3. These 10 parenchymal abnormalities, including their subcategories, were evaluated and scored according to a previously reported chest CT scoring system (Table 1).[15, 17] We calculated the total and the subtotal scores as the sum of each subcategory of bronchiectasis, cellular bronchiolitis, consolidation, and nodule. We also recorded the absolute number of segments showing bronchiectasis, cellular bronchiolitis, consolidation, or nodule involvement. Average of the two radiologists’ scores was used for statistical analysis.

**Table 1:** Computed tomography (CT) scoring system for assessment of *Mycobacterium avium* complex (MAC) disease extent.

	Score			
CT Findings (Maximum Score Possible)	0 Points	1 Point	2 Points	3 Points
Bronchiectasis (9 points)				
Severity*	Absent	Mild	Moderate	Severe
Extent, no. of involved segments	Absent	1–5	6–9	>9
Mucus plugging, no. of involved segments	Absent	1–5	6–9	>9
Cellular bronchiolitis (6 points)				
Severity†	Absent	Mild	Moderate	Severe
Extent, no. of involved segments	Absent	1–5	6–9	>9
Cavity (9 points)	Absent			
Diameter, cm	Absent	<3	3–5	>5
Wall thickness, mm	Absent	<1	1–5	>5
Extent, no. of cavities	Absent	1–3	4–5	>5
Nodule extent, no. of involved segments(3 points)	Absent	1–5	6–9	>9
Consolidation extent, no. of involved segments(3 points)	Absent	<3	3–5	>5

*Mild = bronchus diameter greater than adjacent vessel diameter; moderate = bronchus diameter two to three times vessel diameter; severe = bronchus diameter greater than three times vessel diameter.

†Mild = peripheral lung, 1 cm from pleura; moderate = involvement greater than 1–3 cm from pleura; severe = extending to central lung.

### Statistical analyses

Categorical variables are summarized as frequency and percent, and continuous variables are presented as mean ± standard deviation (SD). The differences in score between initial and follow-up chest CTs were evaluated using the paired t-test and the Wilcoxon signed rank test.

To evaluate which parenchymal abnormalities progress most frequently, we performed repeated measures analysis of variance (ANOVA), and Mauchly’s test was used to test the assumption of sphericity. Because our study violated this assumption, the Greenhouse-Geisser correction was used to adjust to the F-test in terms of degrees of freedom. The interobserver agreements on each subtotal score of bronchiectasis, cellular bronchiolitis, consolidation, and nodule in both initial and follow-up CT studies were assessed by intraclass correlation coefficient (ICC). The ICC value was interpreted as follows: 0–0.2, poor; 0.3–0.4, fair; 0.5–0.6, moderate; 0.7–0.8, strong; > 0.8, almost perfect. All statistical analyses were performed using SPSS version 23.0 (SPSS Inc., Armonk, NY, USA); p<0.05 indicated statistical significance.

## Results

### Patient characteristics

Table 2 shows the characteristics of 40 patients with the nodular BE form of MAC lung disease. Among the 40 patients, 33 (82.5%) were female, the mean age was 64.2 ± 9.4 years, and the mean BMI was 20.9 ± 2.5 kg/m^2^ at the time of diagnosis. Regarding etiology, *Mycobacterium intracellulare* accounted for 20 cases (50%), *Mycobacterium avium* accounted for 15 (37.5%) cases, and both organisms were the etiological cause of 5 cases (12.5%). Positive acid fast staining (AFB) smear rate was 17.5% All of the patients had at least one respiratory symptom, such as cough (n = 31, 77.5%), sputum (n = 31, 77.5%), hemoptysis (n = 11, 27.5%), or dyspnea (n = 5, 12.5%). Diabetes mellitus and malignancy were the most common comorbidities. The mean interval between the initial and last follow-up chest CT scan was 6.23 ± 1.95 years. After 4 years of follow-up periods, 15 of 40 patients received treatment.

**Table 2:** Characteristics of 40 patients with the nodular bronchiectatic form of *Mycobacterium avium* complex lung disease.

Baseline characteristics (n = 40)	
Age at diagnosis (mean ± SD, year)	64.20 ± 9.43
Sex, female	33 (82.5)
BMI (mean ± SD, kg/m^2^)	21.40 ± 2.80
Smoking history	
Ever smoker	5 (12.5)
Non-smoker	35 (87.5)
Duration of CT follow-up (mean ± SD, year)	6.23 ± 1.95
Positive AFB smear	7 (17.5)
Etiologic organisms	
*Mycobacterium intracellulare*	28 (70)
*Mycobacterium avium*	8 (20)
Both	4 (10)
Respiratory symptoms	
Cough	31 (77.5)
Sputum	31 (77.5)
Hemoptysis	11 (27.5)
Dyspnea	5 (12.5)
Medical history	
DM	2 (5.0)
Chronic liver disease	1 (2.5)
Rheumatic disease	1 (2.5)
Malignancy	2 (5.0)

Data are presented as mean ± standard deviation or the number (%) of subjects.

SD- standard deviation; BMI- body mass index; CT- computed tomography; AFB-acid fast bacilli; DM- diabetes mellitus

### Interval changes in CT findings

The strength of interobserver agreement on five CT features of initial and follow-upper studies ranged strong to almost perfect (the ICC value range, 0.710–0.981), except the agreement on the nodule in the initial study (Table 3). During the follow-up period, 39 patients (97.5%) experienced an increase in CT score, while 1 (2.5%) had no change. We observed score increases for bronchiectasis in 33 patients (82.5%), cellular bronchiolitis in 28 patients (70.0%), cavities in 24 patients (60.0%), consolidation in 27 patients (67.5%), and nodules in 16 patients (40%). The total score on follow-up CT increased by a mean 4.9 points compared with initial CT (overall difference = 4.89, p<0.001). The mean final follow-up CT score for all five parenchymal abnormalities increased significantly from the initial CT score. When we compared within each of the 10 subcategories, all mean final follow-up CT scores, except mucus plugging, increased statistically from the initial CT scores (Table 4).

**Table 3:** Interobserver reliability for each radiologic findings of MAC lung disease assessd by interclass correlation coefficient.

	Initial (0)		Follow up CT (1)	
	ICC	95% CI	ICC	95% CI
Bronchiectasis	0.917	(0.843, 0.956)	0.930	(0.868, 0.963)
Cellular bronchiolitis	0.866	(0.747, 0.929)	0.896	(0.803, 0.945)
Cavity	0.968	(0.939, 0.983)	0.981	(0.965, 0.990)
Nodule	-0.111*	(-1.101, 0.412) *	0.710	(0.451, 0.846)
Consolidation	0.847	(0.711, 0.919)	0.919	(0.846, 0.957)
Total	0.957	(0.919, 0.977)	0.972	(0.947, 0.985)

ICC- interclass correlation coefficient

* In this parameter, 36 patients were scored as 0 and the remaining 4 patients were scored as either 0 or 1 by two observer.

**Table 4:** Comparison of CT scores of 10 parenchymal abnormalities between initial and follow-up CT in 40 patients with MAC lung disease.

	Initial	Follow-up	p-value
Bronchiectasis	3.46 ± 1.38	4.49 ± 1.27	<0.001
Severity	1.41 ± 0.56	1.81 ± 0.73	<0.001
Extent	1.74 ± 0.71	2.35 ± 0.62	<0.001
Mucous plugging	0.31 ± 0.60	0.33 ± 0.51	0.860
Cellular bronchiolitis	4.21 ±0.87	4.98 ±0.70	<0.001
Severity	2.03 ± 0.39	2.23 ± 0.47	0.008
Extent	2.19 ± 0.67	2.735± 0.42	<0.001
Cavity	0.99 ± 1.87	3.15 ± 2.61	<0.001
Diameter	0.26 ± 0.49	0.73 ± 0.63	<0.001
Extent	0.29 ± 0.57	0.99 ± 0.99	<0.001
Wall thickness	0.44 ± 0.84	1.44 ± 1.18	<0.001
Nodules, extent	0.05± 0.15	0.34± 0.41	<0.001
Consolidation extents	0.79± 0.66	1.44 ± 0.88	<0.001
Total score	9.50 ± 3.24	14.39± 3.57	<0.001

All numbers are expressed as mean ± standard deviation

When analyzing the five parenchymal abnormalities of each CT pattern, there were significant differences in total score (ANOVA; F = 13.253, p<0.001). In post hoc pairwise comparisons, cavity showed a significant difference compared with cellular bronchiolitis (mean difference = 1.400, 95% CI: 0.203–2.597, p = 0.013), nodules (mean difference = 1.875, 95% CI: 0.683–3.067, p<0.001), and consolidation (mean difference = 1.513, 95% CI: 0.343–2.682, p = 0.004). However, there was no significant difference in mean score change between cavity and bronchiectasis (p = 0.073).

**Fig 4:**
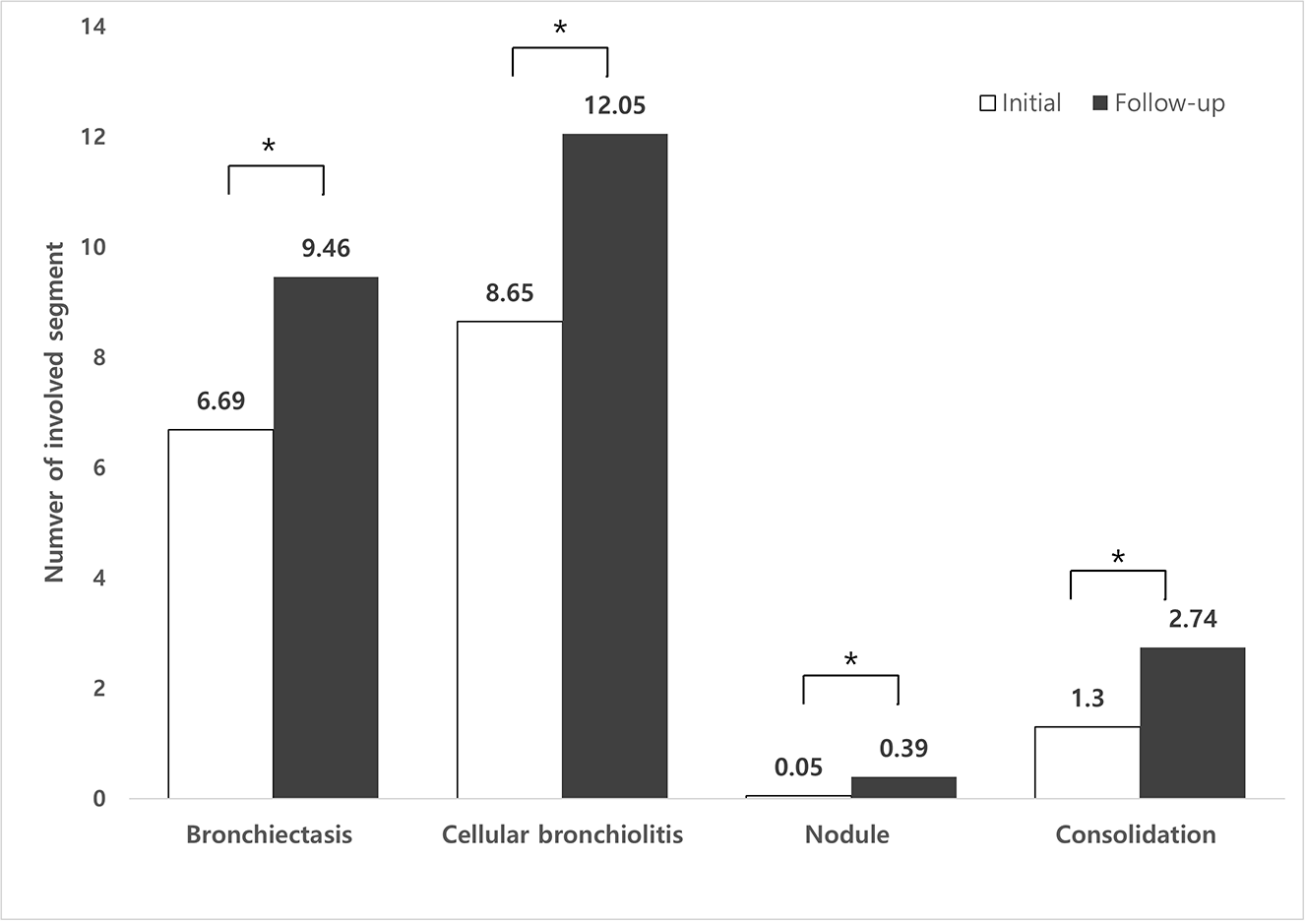
The change of number of involved segment in each radiologic findings. Asterisks indicate statistically significant differences (p<0.001).

Additionally, we investigated the correlations between four parameters (bronchiectasis, cellular bronchiolitis, nodule and consolidation) and the absolute number of involved segments. Four parameters increased significantly in involved segments during the follow-up period (p<0.001) (Fig 4). When analyzing the four parameters of each CT pattern, there were significant increases in involved segment (ANOVA; F = 13.253, p<0.001). In post hoc pairwise comparisons, bronchiectasis showed a largest increase compared with nodules (mean difference = 2.438, 95% CI: 1.316–3.559, p<0.001) and consolidation (mean difference = 1.338, 95% CI: 0.349–2.326, p<0.003). However, there was no significant difference between bronchiectasis and cellular bronchiolitis (p = 0.850).

## Discussion

The consensus recommendation for the nodular BE form of MAC lung disease is long-term follow-up without immediate treatment because it progresses slowly over a long period. This recommendation was supported by clinical and radiologic studies that demonstrated that MAC lung disease is characterized by slow progression[12, 18]. However, herein, although all patients did not received treatment due to minimal or mild symptoms, 97.5% of patients showed an increased in mean CT score over the follow-up period. Furthermore, bronchiectasis and cavity were associated with disease progression.

It is known that the nodular BE form of MAC lung disease progresses particularly slowly over time. In addition, half of these patients remained stable without treatment, and the clinician did not start treatment at the time of diagnosis. One observational study reported that 48% of nodular BE form of MAC patients had disease that progressed to the point of requiring treatment during a 32-month follow-up period. [14] Another study showed that half of nodular BE form of MAC lung disease patients experienced disease aggravation during five-years of follow-up. The recent study, which is investigated the natural course of MAC lung disease, indicates that 305 patients (62.5%) showed progressive disease course resulting in treatment initiation within 3 years of diagnosis and only 115 patients (23.6%) exhibited stable MAC lung disease. [19]. However, these studies only evaluated poor prognostic parameters and outcome differences according to treatment, and individual clinicians made treatment decisions without common guidelines. For these reasons, we lack a complete understanding of the long-term natural course of nodular BE form of untreated MAC lung disease. In our study, we included 40 patients who did not treated at least 4 years due to minimal symptoms. According to the recent study, our study populations could be defined as stationary group. However, we found that 97.5% of individuals showed an increase in mean CT score over time, regardless of their symptoms. In particular, five parenchymal abnormalities showed an increase in mean CT score compared with initial CT score. These findings suggest that even though patients were stationary, most of the nodular BE form of MAC lung cases eventually progress if appropriate treatment is not administered.

Characteristic findings of MAC lung disease include bronchiectasis, cavity, cellular bronchiolitis, consolidation, and nodules. Some of these parameters, especially cellular bronchiolitis, are reversible after treatment.[20] However, cavity and bronchiectasis rarely improve after treatment.[21] Therefore, it is important to determine the parameters that progress more rapidly than others. To investigate this, we conducted pairwise comparisons and a correlation analysis of the relationships between observation time and radiologic changes of five parameters. We found that cavity showed the largest increase of score on follow-up chest CT. Additionally, bronchiectasis showed the largest increase of involved segment compared with nodules and consolidation. The known risk factors of treatment failure are low BMI, atelectasis, cavity, and bronchiectasis.[20, 22, 23] In addition, delayed treatment is also a risk factor of relapse.[24] This finding is important because, in this study, we found that cavity and bronchiectasis progressed over a long-term follow-up period, albeit usually slowly. Generally, there are no guidelines for when treatment should be started, so clinicians decide independently whether to treat depending on patients’ symptoms and changes in radiologic findings. However, we found that those who have MAC lung disease without treatment due to minimal symptoms also lead to radiologic deterioration on chest CT during long-term follow-up period, especially bronchiectasis and cavity. Simple chest PA could not detect bronchiectasis and small cavitary lesion. Therefore, careful CT monitoring with appropriate interval might be beneficial even if patients have minimal symptoms. However, we also need to consider a risk of radiation exposure when performing serial CT. Further study is needed on appropriate follow-up interval of serial CT scans.

The causes of bronchiectasis in nodular BE form of MAC lung disease are uncertain. Usually, MAC lung disease is common among patients with bronchiectasis; this could be because MAC lung disease is also common among cystic fibrosis patients.[25] However, according to our study, MAC infection could also cause bronchiectasis. We found that bronchiectasis developed in normal lungs over long-term follow-up periods, and some patients developed bronchiectasis in previously normal lobes. Our hypothesis is that recurrent or chronic airway infection may cause airway remodeling and ultimately lead to permanent airway dilatation-bronchiectasis. These structural abnormalities could induce poor mucociliary clearance, which instigates continued chronic infection, perpetuating the vicious cycle.[8, 26] One study also reported that the destruction of bronchial structure caused by extensive granuloma formation throughout the airway can lead to bronchiectasis. [27]

Our study has several limitations. First, the small sample size and retrospective design may decrease the statistical power of the findings. Nevertheless, this study is valuable because it is the first to investigate the long-term natural course of untreated cases of the nodular BE form of MAC lung disease by analyzing follow-up chest CT. Second, we were able to analyze only initial and final CT data, not multiple CT data across the follow-up period. This makes it difficult to evaluate sequential morphological aggravations. Finally, we used different CT scanners due to long-term follow-up periods. Therefore, sometimes, we could overestimate or underestimate the parameters according to the CT scanners. Despite these limitations, this study describes the long-term outcome of untreated MAC lung disease in stationary group. A thorough understanding of the natural course of untreated MAC infection will be of substantial help to clinicians.

## Conclusions

Patients with the nodular BE form of MAC lung disease showed aggravation on chest CT after long-term observation. Among 5 CT parameters, the score of cavity tended to increase more than other parameters except bronchiectasis. In addition, bronchiectasis showed more increase in involved segment compared with nodule and consolidation. Considering that bronchiectasis and cavity are not reversible conditions and can lead to poor long-term prognosis, appropriate CT follow-up for the nodular BE form of MAC lung disease could be considered.
